# Zebrafish (*Danio rerio*) Embryo–Larvae as a Biosensor for Water Quality Assessment

**DOI:** 10.3390/biology14111533

**Published:** 2025-10-31

**Authors:** María Santos-Villadangos, Vanesa Robles, David G. Valcarce

**Affiliations:** Cell Biology Area, Molecular Biology Department, Universidad de León, Campus de Vegazana s/n, 24071 León, Spain; msantv03@estudiantes.unileon.es

**Keywords:** WWTP, influent, effluent, zebrafish, biosentinel

## Abstract

**Simple Summary:**

Wastewater treatment plants (WWTPs) are crucial for reducing pollutants and safeguarding ecosystems and human health. This study evaluated the quality of influent water (treated water before secondary (biological) treatment) and effluent water (discharged water after secondary treatment) from the León (Spain) WWTP. We used zebrafish (*Danio rerio*) embryos and larvae as sentinel organisms. Larvae were exposed to different concentrations of influent and effluent during their first 120 h of development, and multiple biological endpoints were analyzed, including survival, hatching, morphology, heart rate, behavior, regeneration, primordial germ cell migration, and gene expression. Exposure to 100% influent caused the strongest effects, including reduced survival, higher number of malformations, decreased heart rate, impaired regeneration, altered behavior and cell migration, and gene expression deregulation. Effluent exposure produced milder effects, decreased further when diluted, reflecting the natural mixing with river water. These results confirm that zebrafish embryos and larvae are sensitive biosensors capable of detecting subtle phenotypic, behavioral, regenerative, and molecular alterations linked to water quality. This research highlights zebrafish as a practical and cost-effective tool to complement current water quality monitoring of effluents released from treatment plants, enhancing the evaluation of effluent safety and contributing to improving environmental and public health protection.

**Abstract:**

Wastewater treatment plants (WWTPs) play a key role in the protection of the environment and public health by reducing the levels of pollutants released into the water. Here, we evaluate the quality of water obtained from two key points of the treatment process of a municipal WWTP (León, Spain) using zebrafish (*Danio rerio*) embryos and larvae as sentinels. Three experimental groups were established: (1) “Control” (CTRL) maintained in embryo medium, (2) “Influent” (I) exposed to influent water before the secondary (biological) treatment (concentrations: I-100% and I-75%), and (3) “Effluent” (E) exposed to effluent water from the secondary treatment (concentrations: E-100% and E-75%). Our results confirmed that survival was subtly affected in I-100% and E-100%, as well as the hatching rate in the effluent. Larvae exposed to both experimental conditions also presented a higher rate of malformations, affecting biometry and showing reduced embryo motility, with the exception of E-75%. The I-100% condition also caused reduced heartbeat, reduced fin regeneration, and a higher number of delocalized primordial germ cells. I-100%-exposed larvae showed dysregulation of four genes (*foxm1l*, *cenpf3b*, *hoxc6a*, and *ddit3*) out of the 19 studied. Effluent dilution mitigated the observed effects, and the model proved to be an effective additional test for wastewater treatment plants.

## 1. Introduction

Wastewater treatment plants (WWTPs) play a key role in protecting public health and the environment by mitigating the negative effects of pollutants released into aquatic ecosystems. However, WWTPs are facing increasingly complex challenges due to the accelerated expansion of urbanization and industrialization [1]. The classification of wastewater is primarily categorized based on its source into municipal, agricultural, or industrial [2]. Typically, WWTPs are designed to treat wastewater efficiently, balancing cost-effectiveness while following water quality standards [3]. The characteristics of influent wastewater, such as pollutant type, concentration, and quantity, must be analyzed to determine WWTP specifications. WWTPs involve the sequential use of various wastewater treatment methods, which are selected by considering many factors, such as the influent wastewater characteristics, regulatory standards, technological availability, and economic viability [4]. A wastewater treatment process is generally classified into four levels: preliminary, primary, secondary, and tertiary, according to the removal of specific pollutants [5,6]. The complexity of treatment required for these levels depends on the source, type, flow, characteristics, and intended use of the wastewater [3]. Preliminary treatment removes large debris, grit, and solids from wastewater through processes such as screening, comminution, and grit removal [4]. Subsequently, primary treatment is employed to extract the remaining solids and organic matter through the application of gravitational forces, using flotation systems, primary sedimentation tanks, neutralization tanks, and equalization tanks to remove suspended solids, grit, fats, and oils through processes such as sedimentation, coagulation, and flocculation [1,4]. Specifically, the European directive concerning urban wastewater treatment (91/271/EEC) states that after primary treatment, the Biochemical Oxygen Demand over five days (BOD_5_) of the incoming wastewater must be reduced by at least 20% before discharge, and the total suspended solids of the incoming wastewater must be reduced by at least 50%. Then, this effluent water undergoes secondary treatment, which involves the implementation of biological techniques to reduce the levels of organic matter, nitrogen, and phosphorus in wastewater through aerobic and anaerobic digestion [2,4], and must fulfill specific values (BOD_5_, nitrogen, and phosphorus, among others) established by the European directive (91/271/EEC). Some of the most efficient WWTPs include tertiary treatment, also known as advanced treatment, which includes methods such as membrane filtration, adsorption, chemical oxidation, and ion exchange [4]. Despite the capacity of these unit processes to guarantee ultra-purity, their implementation is limited by the high cost and the absence of expertise [7] and may not be necessary in all cases. In particular, León (Spain) WWTP —the focus of our study—does not have tertiary treatment, although its BOD_5_ elimination performance is satisfactory and it meets legal standards at the national and European level.

Further, the measurement of all parameters in the effluent is a time-consuming process that requires the use of complex tests and hazardous materials. To address these challenges, recent advancements in electrical sensor technologies have enabled real-time monitoring of various effluent quality parameters. Nevertheless, key indicators such as BOD_5_ and Chemical Oxygen Demand (COD) remain difficult and expensive to quantify using sensor-based methods [8]. Other approaches to determine the quality of the effluent water from WWTPs include the implementation of Decision Support Systems (DSSs), which are designed to assist users in efficiently identifying optimal solutions [1], and the integration of artificial intelligence techniques, which have emerged in recent years as revolutionary tools in this domain [8]. In parallel, biological approaches using fish as bioindicators have gained increasing relevance for evaluating the ecological and physiological impacts of WWTP effluents. Several studies on fish species have analyzed the effects of WWTP effluents using diverse biological endpoints, including gene expression [9], immune function [10], and ecological indicators. On one hand, field studies have reported significant differences in fish abundance, biomass, and spatial distribution between upstream and downstream areas near WWTP discharges [11], showing the importance of evaluating these waters. On the other hand, bioaccumulation of pharmaceuticals [12] has been documented in several wild fish, demonstrating their environmental persistence and potential to disrupt the endocrine system as well as the immune system, as reported in a study on European seabass (*Dicentrarchus labrax*) [10]. Some of these studies have also demonstrated that fish can serve as sensitive biological indicators, as observed reductions in adverse biological responses have corresponded with improvements in effluent quality following upgrades in WWTP infrastructure [9].

In the present work, we use zebrafish (*Danio rerio*) as a model species to study water quality instead of other fish species due to its multiple advantages. This species is widely used as a model in many research fields such as ecotoxicology [13,14,15], neuroscience [16], genetics [17], development [18], and biomedicine [19], among others. Some of its advantages as an experimental model include its ease of maintenance and storage as well as high prolificacy. Besides the fact that it has approximately 70% orthologous genes with humans [20], its rapid embryogenesis and optical transparency in its embryonic and larval stages enable the monitoring of the expression of target genes using specific molecular markers [21,22]. This species also represents a valuable model system, given the wide availability of mutant and transgenic lines accessible to the scientific community [15]. In this work, we used two zebrafish transgenic lines: *kop Tg(kop:mScarlet-I-nos 3′UTR-cmlc:GFP)* [23] and *hsp70 Tg(hsp70l:dn-fgfr1a-EGFP)* [24,25]. The first line allows us to visualize the heart and the primordial germ cells (PGCs), which are the embryonic precursors of gametes and migrate within the embryo until they reach the genital ridge [26], while the second emits green fluorescence and provides a particular phenotype in response to cellular stress. We propose using the zebrafish model as a promising, cost-effective, and accessible alternative to current wastewater analysis methods due to its advantageous characteristics. Thus, the implementation of its use as an in vivo evaluation tool will potentially enhance WWTP operations, offering solutions to complex challenges and contributing to the development of a more sustainable and efficient wastewater treatment infrastructure.

While previous studies have demonstrated the benefits of zebrafish as an ecotoxicology model, our research aims to provide novel insights by focusing specifically on the first 120 h post-fertilization (hpf), since current EU legislation (European Directive 2010/63/EU) does not apply to zebrafish specimens until they are over this age, once the species’ organogenesis is complete and larvae are autonomous in feeding and have a functioning digestive system [27].

We hypothesize that the zebrafish embryo-larvae model functions as a sensitive biosensor for water quality, capable of detecting molecular, phenotypic, behavioral, and regenerative alterations associated with different stages of water treatment. Therefore, our main objective is to develop a useful tool for water quality analysis that can be implemented as an additional test in WWTPs. For this purpose, our specific objectives include the analysis of a range of developmental outcomes, including survival, hatching, malformations, biometric parameters, heart rates, PGCs migration, behavior, fin regeneration, and gene expression. In addition, to better approximate real environmental conditions, we also aim to evaluate the effects of diluted effluent, as it would ultimately mix with river water after discharge.

## 2. Materials and Methods

### 2.1. Ethics

All protocols used in this study were carried out in accordance with Spanish (RD 53/2013) and European legislation (European Directive 2010/63/EU). No specimen included in our experiments exceeded 120 hpf. Thus, following European legislation (European directive 2010/63/EU), no concrete permission was required.

### 2.2. Animal Maintenance

Adult zebrafish breeders (*Danio rerio*; *kop*—*Tg(kop:mScarlet-I-nos 3′UTR-cmlc:GFP)*—and *hsp70*—*Tg(hsp70l:dn-fgfr1a-EGFP*)) were maintained under standard conditions [28] at the facilities of the Animal Research and Welfare Service of the University of León (Spain). Progenies from routine crossings (1 ♂:2 ♀) [28] were kept in embryo medium (EM) (0.137 M NaCl; 5.4 mM KCl; 0.25 mM Na_2_HPO_4_; 0.44 mM KH_2_PO_4_; 6.5 mM CaCl_2_; 4.99 mM MgSO_4_-7H_2_O: 4.2 mM NaHCO_3_; 50 µL 1% (*w*/*v*) methylene blue/L) [28] in Petri dishes until splitting into experimental replicates.

### 2.3. Experimental Design

**Figure 1:**
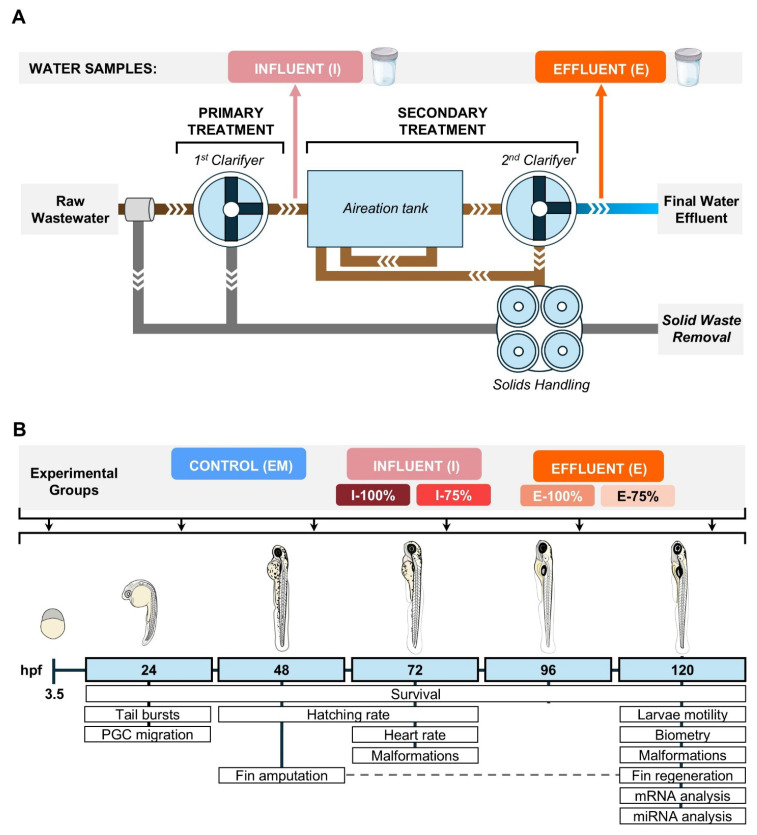
Experimental design. (**A**) Schematic diagram of the León Wastewater Treatment Plant (WWTP). Sampling points before and after secondary treatment are labeled as Influent (I) and Effluent (E). (**B**) Experimental timeline indicating the analyzed parameters at each point of development. hpf: hours post-fertilization.

The experiment involved the first 120 hpf. Three experimental groups were established: (1) “Control” (CTRL)—maintained in EM; (2) “Influent” (I)—exposed to different concentrations of influent water to the secondary treatment in the León WWTP (I-100% and I-75%); and (3) “Effluent” (E)—maintained in different concentrations of effluent water from the secondary treatment in the León WWTP (E-100% and E-75%) (Figure 1A). Each biological replicate consisted of 30 canonically developed embryos [21] at 3.5 hpf maintained in 8 mL of EM or experimental dilution under standard culture conditions (Figure 1B). All experiments were carried out with *kop* specimens except for the dose–effect trial, in which we used *hsp70* fish.

### 2.4. Progeny Evaluation

#### 2.4.1. Survival, Hatching, and Malformation Evaluation

Survival was studied daily (Figure 1B). At 48 and 72 hpf, the hatching rate (hatched embryos/number of live specimens) was calculated. The malformation rate and the type of malformations were also evaluated at 72 and 120 hpf, concurring, respectively, with the end of embryogenesis and end of organogenesis. For image acquisition, we used a Nikon SMZ25 stereomicroscope (Nikon DS-Ri2 camera) (Nikon, Tokyo, Japan). NIS-Elements Advanced Research software (version 6.10.01, Nikon) was used for image processing. The same equipment and software were also used in the remaining experiments.

#### 2.4.2. Heartbeat Analysis

Fluorescence heart videos of four 72 hpf *kop* specimens of each plate (*n* = 56) were recorded for 15 s. Each heart was assigned to a region of interest (ROI) in the clip and processed using NIS-Elements software. The heartbeat was quantified by measuring fluorescence peaks on the ROI-derived histograms generated by the software. The accuracy of this methodology was validated and optimized before conducting final experiments.

#### 2.4.3. PGC Number and Migration

The number of fluorescent PGCs and their correct migration to the genital ridge were evaluated at 24 hpf following previous works [29].

#### 2.4.4. Biometry Analysis

At 120 hpf, 10 larvae of three different plates per condition (*n* = 30) were placed laterally in molds [30] to measure larvae length (LL), yolk sac area (YSA), cardiac area (CA), mouth-to-anus length (MAL), and spine length (SL), as shown in Figure 5E. NIS-Elements software and ImageJ, (version 1.54, Bethesda, MD, USA) were used for quantification.

### 2.5. Behavioral Analysis

Regarding embryo behavior, 1 min clips of 24 hpf embryos (*n* = 30) from each group were recorded. The number of tail burst movements inside the chorion per minute was assessed. At 120 hpf, we recorded the plates top-down (*n* = 15). Larval activity was calculated by quantifying the percentage of active specimens during the 6 min/number of live specimens.

### 2.6. Fin Regeneration Analysis

To evaluate how experimental waters might affect tissue regeneration, we amputated the caudal fin of randomly selected 48 hpf larvae from CTRL, I-100%, and E-100% replicates (*n* = 9) following previous protocols [31]. Larvae were anesthetized (MS222-0.000605 M; Acros Organics, Geel, Belgium), and the fin was cut off, establishing the end of the notochord as a reference. After amputation, larvae were maintained in EM to prevent wound infections until 120 hpf, when we measured the regenerated fin with Adobe Photoshop CS3 software (version 10.0, Adobe Systems Incorporated, San Jose, CA, USA).

### 2.7. Molecular Studies

#### 2.7.1. Sample Collection

At 120 hpf, larvae were euthanized (MS222-0.015 M; Acros Organics, Geel, Belgium) [28]. After washing with PBS 1× (0.8% NaCl; 0.02% KCl; 0.02 M PO_4_), larvae pools from each plate were stored at −80 °C in 150 μL of RNAlater (Invitrogen, Vilnius, Lithuania) until processing.

#### 2.7.2. RNA Extraction

RNA extraction from 6 replicates/condition was performed using the miRNeasy Tissue/Cells Advanced Mini Kit (Qiagen, Hilden, Germany) following the company’s protocol. Quantification, purity, and integrity of extracted RNA were assessed.

#### 2.7.3. Retrotranscription

For each RNA sample, we performed retrotranscription reactions (1 μg of total RNA) using the High-Capacity RNA to cDNA kit (Applied Biosystems, Vilnius, Lithuania) following the manufacturer’s protocol.

#### 2.7.4. Quantitative PCR

qPCR was performed under standard cycling conditions and quality guidelines in a Quant Studio 1 unit (Applied Biosystems, South San Francisco, CA, USA).

The primers used for each studied mRNA are listed in Table 1. *actb2* and *rps18* were used as housekeeping genes.

### 2.8. Dose–Effect Analysis

To evaluate when the effects of the effluent water fade, the *hsp70* transgenic line was used as a reporter. In addition to the five groups previously described (CTRL, I-100%, I-75%, E-100%, and E-75%), we added more effluent dilutions (E-50%, E-25%, E-10%, and E-5%) and included a “Positive control” (C^+^) maintained in EM and subjected to a heat shock (38 °C) for 2 h. The C^+^ plates were placed in a Memmert INE400 incubator (Memmert GmbH + Co.KG, Schwabach, Germany) programmed at 38 °C from 5 to 7 hpf. Each biological replicate consisted of 8 embryos (*n* = 8) per well (24-well plate) maintained in 2 mL of EM to maintain the wastewater/EM ratio.

At 24 hpf, the malformation rate and behavior were evaluated as described above.

### 2.9. Statistical Analysis

All data were analyzed and plotted with GraphPad Prism 8.0.1 software (GraphPad Software, San Diego, CA, USA) except for spider chart plots, which were created using Flourish Software [39]. Survival curve comparison was analyzed using the Mantel–Cox test. The Shapiro–Wilk test was carried out to test normality. When normal variables were compared, a one-way ANOVA was used, while non-parametric variables were compared using a Krustal–Wallis test. Dunnett’s (parametric) and Dunn’s (non-parametric variables) post hoc tests were used to compare each experimental group to the control. Error bars represent the mean ± standard error of the mean (SEM). *p* values < 0.0500 were considered statistically significant.

## 3. Results

### 3.1. Progeny Evaluation

#### 3.1.1. Survival, Hatching, and Malformation Evaluation

**Figure 2:**
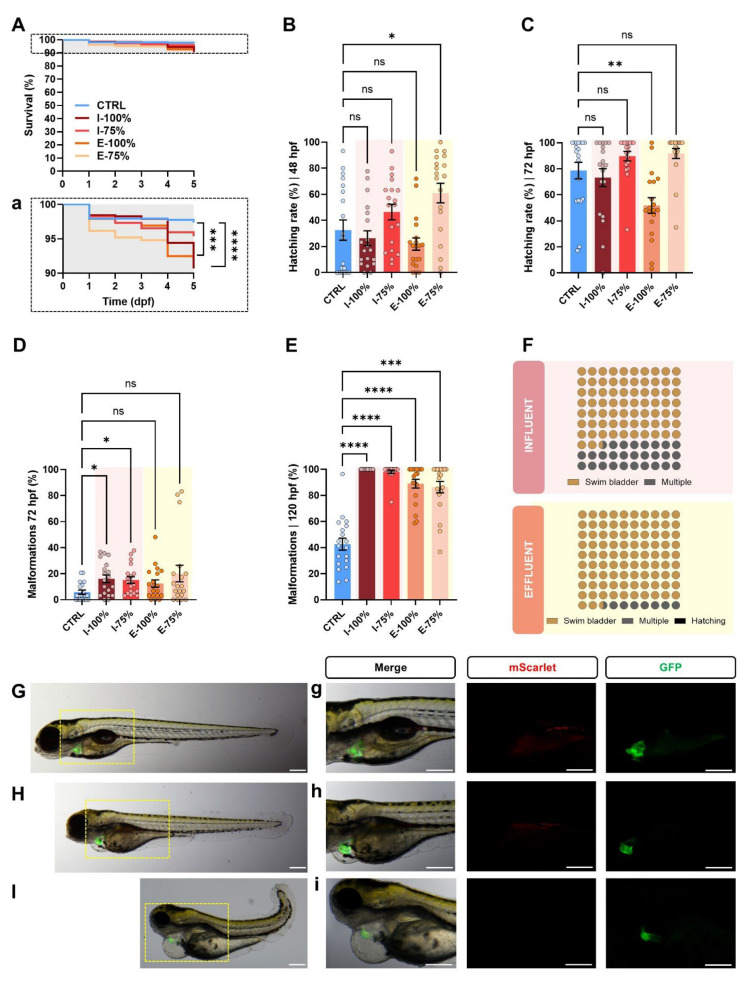
Zebrafish progeny development analysis. (**A**) Kaplan–Meier survival curves (Mantel–Cox test) of the progenies during the experiment. (**a**) Rescaled graph of the dotted area in A for better interpretation of the results. (**B**) Hatching rate (%) at 48 hpf and (**C**) at 72 hpf. (**D**) Malformation rate (%) at 72 hpf and (**E**) at 120 hpf. (**F**) Types of malformations (%) found in influent and effluent larvae at 120 hpf. (**G**–**I**) Extended depth focus images of examples of larvae found in the experiment. (**G**) Control larva at 120 hpf showing a canonical phenotype at this stage. (**H**,**I**) Examples of malformed larvae from the influent and effluent groups at 120 hpf. Both larvae present malformed swim bladder, poor yolk sac reabsorption, yolk sac and pericardial edema, and heart malformations. The specimen in (**I**) presents a more severe phenotype with the addition of skeletal malformation and the absence of PGCs. (**g**–**i**) show the heart and PGC region magnified and their fluorescence. Biological replicates (*n* = 30 initial embryos/plate). CTRL: fish maintained in embryo medium; I-100% and I-75%: fish maintained in 100% and 75% influent water to the secondary treatment in the León WWTP; E-100% and E-75%: fish maintained in 100% and 75% effluent water. The scale of the images is 250 µm. Bars in (**B**–**E**) show the mean value ± SEM (*n* = 19). hpf: hours post-fertilization. dpf: days post-fertilization. * *p* < 0.0500. ** *p* < 0.0100. *** *p* < 0.0010. **** *p* < 0.0001. ‘ns’: not statistically significant (*p* > 0.0500).

Kaplan–Meier curves showed statistically significant differences between CTRL and I-100% (*p* < 0.0001) and E-100% (*p* = 0.0002) groups, with a lower survival rate in the former (Figure 2A). Our survival curve also shows lower mortality in the E-100% group than the I-100% group, proving the toxin removal efficacy in the León WWTP.

Regarding hatching, at 48 hpf, differences between the CTRL and E-75% condition were found (*p* = 0.0142), showing an increase in the ratio of hatched individuals in this effluent water (Figure 2B). At 72 hpf, the only statistically significant decrease was found in E-100% (*p* = 0.0080) (Figure 2C). Although our experiments only revealed significant differences at these two points, there seems to be a tendency for both influent and effluent at 100% to reduce the hatching rate, which fades when the waters are diluted.

In terms of malformations, at 72 hpf, statistically significant differences were observed between CTRL and influent larvae (*p* = 0.0246 and *p* = 0.0246, 100%, and 75%, respectively; Figure 2D). At 120 hpf, a statistically significant increase in malformation rate was reported in all WWTP conditions compared to the control (I-100%, I-75%, and E-100%: *p* < 0.0001; E-75%: *p* = 0.0002). I and E plates showed approximately 2–2.5 times higher mean values than their CTRL counterparts (Figure 2E). The type of malformations found in the influent and effluent groups were absence or very reduced volume of the swim bladder (75.5% and 92.7% of total malformed larvae, I and E, respectively), followed by severe phenotypes involving multiple malformations (27.5% and 7.1%, I and E, respectively), and hatching malformations (0.2% in the effluent group) (Figure 2F).

Examples of the main registered phenotypes are represented in Figure 2G–I. While the control larva shows a canonical development (Figure 2G), the malformed larvae of the influent and effluent group (Figure 2H,I) showed aberrant morphologies, including reduced volume of the swim bladder, poor yolk resorption, cardiac malformations, formation of body and/or pericardial edema, and skeletal malformations. The most severe cases were affected by multiple malformations.

#### 3.1.2. Heartbeat Analysis

**Figure 3:**
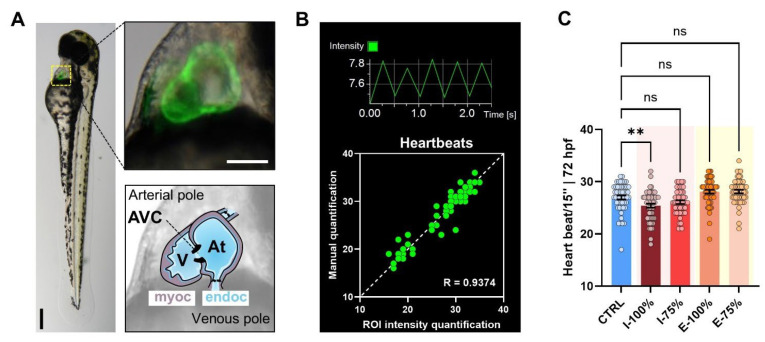
Heartbeat analysis. (**A**) Extended depth focus merge image of a 72 hpf *kop* (*kop:mScarlet-I-nos 3′UTR-cmlc:GFP*) specimen showing the fluorescent heart. AVC: atrioventricular canal; V: ventricle; At: atrium; myoc: myocardium; endoc: endocardium. The scale of the images is 250 µm. (**B**) Example of a histogram registering the GFP fluorescence intensity from the hearts and correlation plot showing the accuracy of the method used compared to manual scoring. (**C**) Heartbeat per 15″ at 72 hpf. Individual values (*n* = 56) are represented. ** *p* < 0.0100. ‘ns’: not statistically significant (*p* > 0.0500).

Our transgenic line (Figure 3A) allowed us to report, by using our optimized fluorescence analysis method (Figure 3B), statistically significant differences in the heartbeat between CTRL and I-100% larvae (*p* = 0.0096), showing a decrease in the influent-exposed larvae (Figure 3C). These results are logical since we found cardiac malformations at 72 hpf that could affect the heartbeat.

#### 3.1.3. PGC Migration to the Genital Ridge

**Figure 4:**
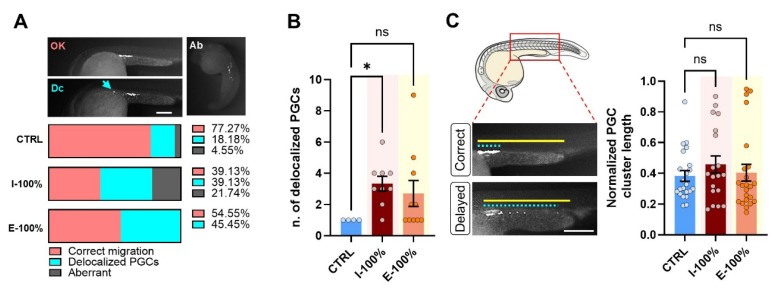
Primordial germ cell analysis. (**A**) Representative greyscale (white: mScarlet) images of 24 hpf *kop* phenotypes found in the experiment: OK refers to correct migration of the PGCs to the genital ridge; Dc refers to an embryo showing a delocalized PGC (blue arrow); and Ab refers to an aberrant example. The percentage graphs displayed below show the percentages of each type within the CTRL, I-100%, and E-100% groups. (**B**) Number of delocalized PGCs in the embryos showing this phenotype. (**C**) Representative images of embryos analyzed for PGC cluster length (quantification shown in plot on the right). The yellow line shows the length of the yolk extension (normalizer), and the dotted blue line the length of the PGC cluster. The scale of the images is 250 µm. * *p* < 0.0500. ‘ns’: not statistically significant (*p* > 0.0500).

Under the stereomicroscope, we found three phenotypes regarding PGC migration (Figure 4A): correct migration dynamics, embryos showing delocalized PGCs, and aberrant embryos. As expected, the highest percentage of aberrant and delocalized phenotypes was found in I-100% individuals. Within those embryos showing PGCs far from their expected niche, the number of delocalized PGCs was statistically significantly higher in the I-100% group (*p* = 0.0194; Figure 4B). However, the in-depth evaluation of the cluster length did not report statistically significant differences (Figure 4C).

#### 3.1.4. Biometry Analysis

**Figure 5:**
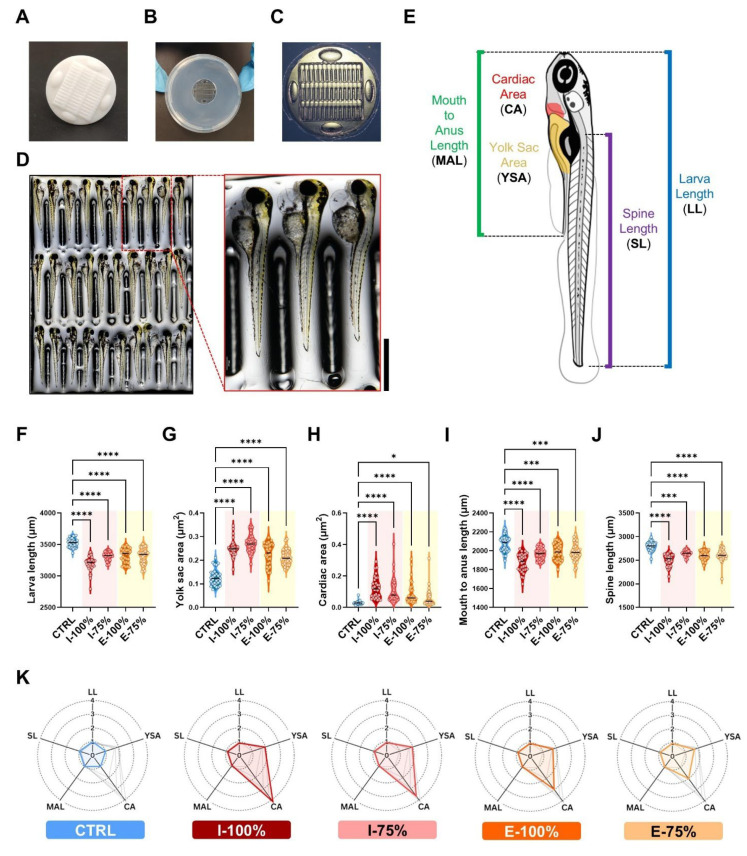
Impact of wastewater on zebrafish biometry at 120 hpf (end of the experiment). Larvae were monitored using a using a 3D-printed stamp. (**A**) Clean stamp surface. (**B**) Ready-to-use 1% agarose mounting cast prepared in a 55 mm Petri dish. (**C**) Mounting cast under the stereomicroscope. (**D**) Representative extended depth focus tiles image (and magnified detail) of a batch of larvae to analyze. The example corresponds to individuals from the I-100% group. Scale bar: 250 μm. (**E**) Description of the analyzed biometry parameters. (**F**–**J**) Violin plots showing individual values (*n* = 30) for each parameter. (**K**) Spider charts representing normalized data (CTRL mean values = 1) for each experimental group. * *p* < 0.0500. *** *p* < 0.0010. **** *p* < 0.0001.

The use of molds (Figure 5A–D) to homogenize the orientation of the fish larvae allowed us to easily quantify the five biometrical parameters studied in this project (Figure 5E). The data showed a significant decrease in the LL between all conditions regarding the control (*p* < 0.0001) (Figure 5F). Inversely, YSA showed a significant increase between all groups compared to the control (*p* < 0.0001) (Figure 5G), likewise for the CA (*p* < 0.0001; E-75% *p* = 0.0276) (Figure 5H). The MAL (*p* < 0.0001; E-100% *p* = 0.0002; E-75% *p* = 0.0004) (Figure 5I) and the SL (*p* < 0.0001; I-75% *p* = 0.0002) (Figure 5J) also reported a statistically significant reduction in their mean values. The normalized data with respect to the control are shown in Figure 5K, where it is noticeable that E-75% shows the most normal spider chart of the wastewater groups.

### 3.2. Behavioral Analysis

**Figure 6:**
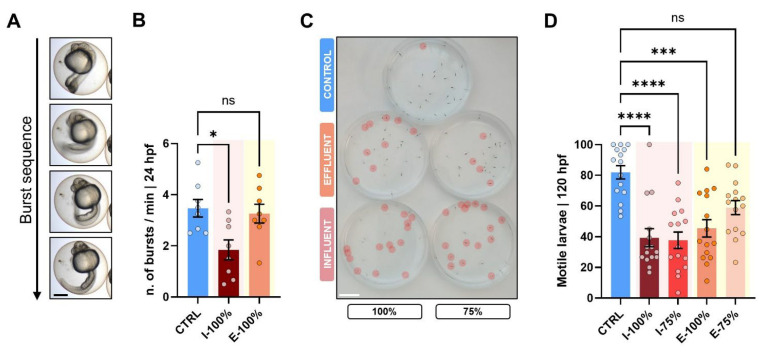
Behavioral analysis. (**A**) Clip sequence showing the events of an embryo burst. (**B**) Number of embryo bursts at 24 hpf within the chorion (*n* = 8). Scale: 250 µm. (**C**) Capture of the videos showing immotile larvae (red circles) at 120 hpf during 6 min of analysis. Scale: 1 cm. (**D**) Motile larvae (%) at 120 hpf. hpf: hours post-fertilization. Mean value ± SEM (*n* = 15) is represented. * *p* < 0.0500. *** *p* < 0.0010. **** *p* < 0.0001. ‘ns’: not statistically significant (*p* > 0.0500).

We analyzed the specimens’ behavior at two key points, 24 hpf and 120 hpf, to evaluate the impact of wastewater. At 24 hpf, we evaluated the number of embryo bursts per minute (Figure 6A), registering a reduced number of movements in the I-100% group (*p* = 0.0407) compared to CTRL (Figure 6B). At 120 hpf, we evaluated the ratio of motile larvae during a 6 min frame (Figure 6C), and we found statistically significant differences (*p* < 0.0001) between the CTRL and the rest of the groups excluding E-75% (*p* = 0.0958) (Figure 6D). I-100%, I-75%, and E-100% larvae registered around 40% mean values of motile larvae in the plates vs. CTRL, scoring a mean of 81.95%.

### 3.3. Fin Regeneration Evaluation

**Figure 7:**
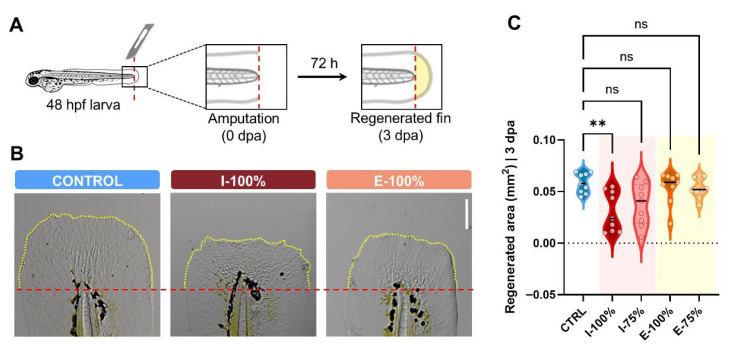
Impact of wastewater on zebrafish regeneration ability. (**A**) Schematic diagram of the experiment workflow. Caudal fins were amputated from anesthetized 48 hpf larvae exposed to experimental conditions, taking the end of the notochord (dotted red line) as a reference. After 72 h (3 dpa), regenerated fins were photographed and the area was quantified. (**B**) Extended depth focus images showing examples of the regenerated caudal fin area. Dotted yellow lines show the margin of the regenerated caudal fin for easier interpretation. (**C**) Quantification of the regenerated area (mm^2^) in each group at 3 dpa. Violin plots show individual values (*n* = 9). hpf: hours post-fertilization. dpa: days post-amputation. ** *p* < 0.0100. ‘ns’: not statistically significant (*p* > 0.0500).

Figure 7A illustrates the procedure followed for caudal fin amputation, including the specific cutting plane and the time window used for regeneration analysis. Representative images of the regenerated fin area under different experimental conditions are shown in Figure 7B, where noticeable differences can be observed. However, the regenerated caudal fin area quantification only showed statistically significant differences between CTRL and I-100% conditions (*p* = 0.0076), while the other conditions did not report any statistically significant differences (Figure 7C). The I-100% group presented a reduced a mean value of 0.0308 ± 0.0065 mm^2^ compared to the CTRL group (0.0584 ± 0.0032 mm^2^). These results indicate that the chemical profile of the influent waters alters regeneration.

### 3.4. Molecular Studies

#### Gene Expression

**Figure 8:**
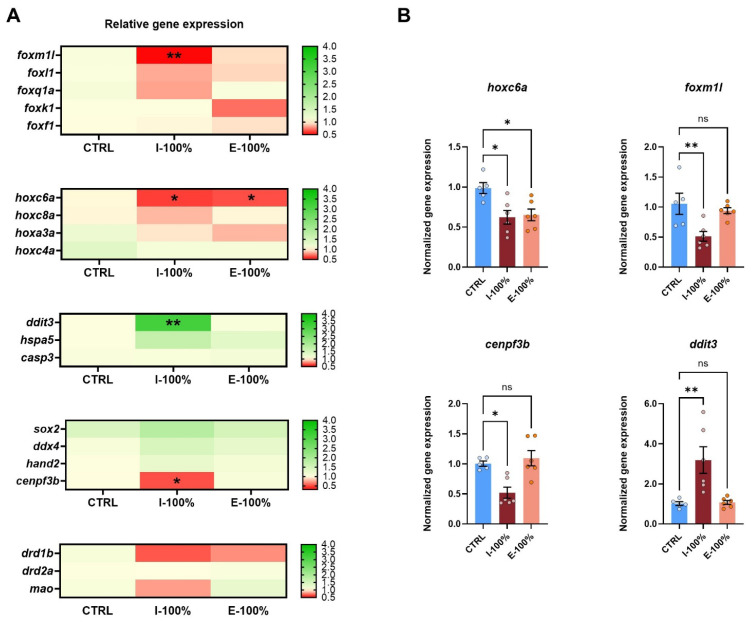
Analysis of 120 hpf larvae gene expression. (**A**) Heatmaps showing the mean normalized gene expression (HKGs: *actb2* and *rps18*) of *fox* genes (*foxf1*, *foxk1*, *foxl1*, *foxm1l*, and *foxq1a*), *hox* genes (*hoxa3a*, *hoxc4a*, *hoxc6a*, and *hoxc8a*), endoplasmic reticulum stress- and apoptosis-related genes (*hspa5*, *ddit3*, and *casp3*)**,** canonical phenotype-related genes (*sox2*, *ddx4*, *hand2*, and *cenpf3b*), and dopamine- and serotonin-pathway-related genes (*drd1b*, *drd2a*, and *mao*). (**B**) Histograms of differentially expressed genes within each batch. (*n* = 6 independent experiments; 20–30 larvae pool per sample). Mean value ± SEM (*n* = 6) is represented. * *p* < 0.0500. ** *p* < 0.0100. ‘ns’: not statistically significant (*p* > 0.0500).

Our gene expression analysis showed clear patterns on the different batches of studied genes (Figure 8A). While the candidate *hox* and *fox* genes showed a global downregulation trend, cellular-stress-related genes reported an upregulation trend, and the group of key genes on development and dopamine and serotonin pathways did not show clear tendencies with the exception of *cenpf3b* and *drd1b*. Our qPCR experiments revealed a statistically significant downregulation of *hoxc6a* in both I-100% (*p* = 0.0236) and E-100% (*p* = 0.0429) experimental conditions (Figure 8B).

We also found a downregulation in *foxm1l* (*p* = 0.0059) and *cenpf3b* (*p* = 0.0347) in the larvae exposed to influent water (Figure 8B), which belong to the *fox* family.

Focusing on the genes related to apoptosis and endoplasmic reticulum (ER) stress, *ddit3* showed a statistically significant (*p* = 0.0051) overexpression (normalized gene expression of 3.191 ± 0.6630) in the I-100% group, whereas *hspa5* and *casp3* gene expression did not report statistically significant differences between the mentioned groups (Figure 8).

### 3.5. Additional Analysis

**Figure 9:**
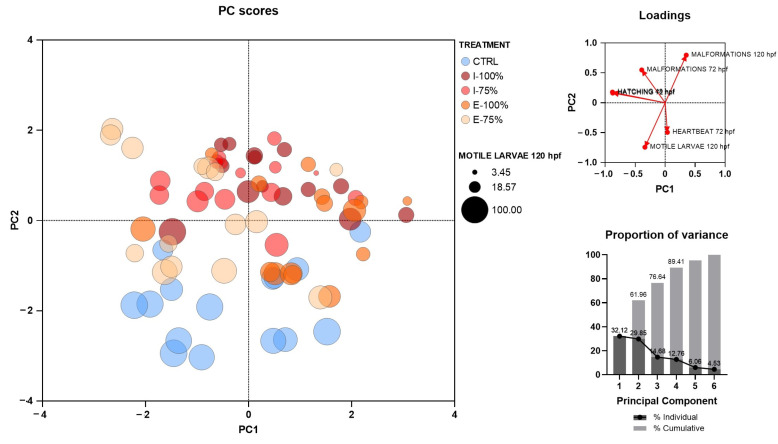
Principal component analysis (PCA) representing individual biological replicates (*n* = 30 initial embryos/dish) exposed to embryo medium (CTRL), 100% (I-100%) and 75% (I-75%) of influent water to the secondary treatment in the León WWTP, and 100% (E-100%) and 75% (E-75%) of effluent water. A cumulative metabolic variance of 82.45% is described by the first three principal components (PCs), with 38.08% by PC1, 27.04% by PC2, and 17.33% by PC3.

After the experiments, advanced statistical analyses were performed on the samples obtained to explore patterns among the biological responses. In particular, a principal component analysis (PCA) was conducted using variables that can be easily assessed at a wastewater treatment plant (WWTP) with basic equipment such as a stereomicroscope: survival, hatching rate, malformation rate, heartbeat, and larval motility. The PCA results revealed three distinct clusters corresponding to control, influent, and effluent samples. Strong associations among the evaluated parameters were identified, with a clear intrinsic relationship between the two hatching times. Malformations also exhibited a consistent pattern across both periods, although they did not fully overlap. Notably, an inverse relationship was observed between the incidence of malformations and both heartbeat rate and larval motility, highlighting their potential as sensitive indicators of developmental stress. Additionally, the first three principal components explained 82.45% of the total variance; this high cumulative variance indicates that these three components effectively capture most of the variability in the dataset, meaning the PCA effectively summarizes the differences between treatment groups and reveals meaningful patterns of association between variables (Figure 9).

### 3.6. Dose–Effect Analysis

**Figure 10:**
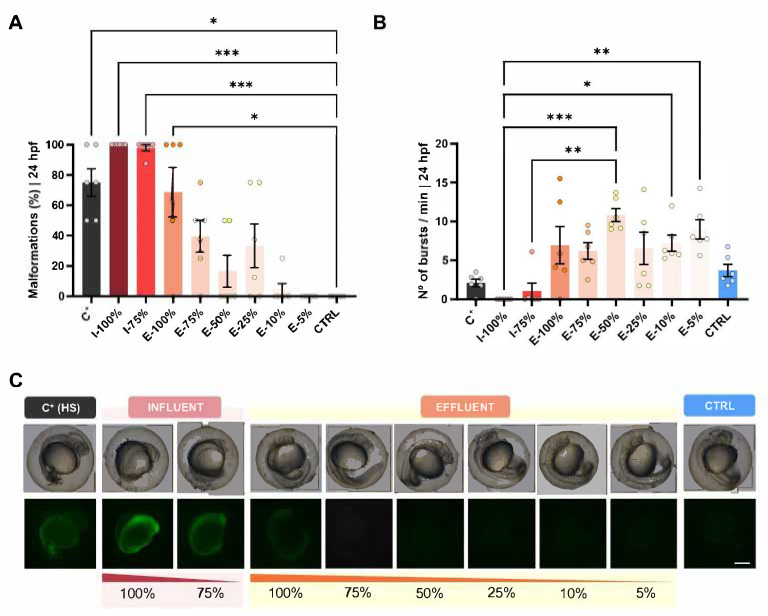
Wastewater dose–effect analysis using the line *hsp70* (*Tg(hsp70l:dn-fgfr1a-EGFP)*) as a positive control. (**A**) Malformation rate (%) at 24 hpf. (**B**) Number of embryo bursts at 24 hpf within the chorion. (**C**) Examples of embryo morphology and fluorescence in each experimental dilution group. The scale of the images is 250 µm. Bars in (**A**,**B**) show mean value ± SEM (*n* = 6). hpf: hours post-fertilization. C^+^: fish maintained in embryo medium and subjected to a heat shock (38 °C from 5 to 7 hpf). * *p* < 0.0500. ** *p* < 0.0100. *** *p* < 0.0010.

In this experiment, we used the *hsp70* transgenic line, as is optimal for toxicity assays. Using this transgenic line, fluorescence was observed at toxic concentrations, consistent with previous reports and coinciding with the appearance of underdeveloped or aberrant phenotypes (Figure 10).

The malformation rate showed significant differences between the CTRL group and the C^+^, I-100%, I-75%, and E-100% groups (*p* = 0.0191, *p* = 0.0003, *p* = 0.0007, and *p* = 0.0387, respectively), showing an increase in malformations (Figure 10A). We also registered statistically significant differences in the amount of 24 hpf embryo tail coiling between the I-100% and E-50% (*p* = 0.0003), E-10% (*p* = 0.0457), and E-5% (*p* = 0.0053) groups and between the I-75% and E-50% conditions (*p* = 0.0046) (Figure 10B). In the number of bursts/min, we can differentiate two different patterns: a lower activity in the influent-exposed larvae and a higher number of bursts in those exposed to the effluent compared to the control. Figure 10C shows the development and morphology of *hsp70* embryos, as well as the presence or absence of fluorescence indicative of a stress response. Embryos exposed to the C^+^ and I conditions exhibit underdeveloped and aberrant phenotypes, accompanied by green fluorescence.

## 4. Discussion

### 4.1. Progeny Evaluation

#### 4.1.1. Survival, Hatching, and Malformation Evaluation

In the present work, we assessed the impact of influent and effluent waters from the León wastewater treatment plant using zebrafish embryos and larvae as biosentinels, as they constitute a widely used model in ecotoxicology research [13,14,15]. Zebrafish are highly sensitive to environmental pollutants and toxins, making them an excellent indicator of water quality by evaluating survival, developmental, morphological, and behavioral parameters, among others [13,40,41,42]. In our study, we reported a statistically significant reduction in the survival curve in I-100% and E-100% compared to CTRL. Despite this, survival remained above 90% across treatments. Similar findings are reported in previous studies with comparable WWTP treatments where mortality did not surpass the 25% or 10% in the studied wastewaters, analogous to I and E waters in our work [40]. Other works studying zebrafish survival curves (5 dpf) also found no differences in WWTP effluent water and its dilutions [43]. Even in WWTP dealing with highly contaminated waters, the tested dilutions I-50% and E-50% showed 45% and 75% survival rates at the end of the experiment (6 dpf), contrary to the lethal I-100% condition [41]. The lethality due to exposure to water influents (I-100%) is recorded in cases where pollution is especially high [13] in plants very far from the León WWTP, which is fed by a small urban center and a relatively unindustrialized surrounding environment. Our data also show lower mortality in E-100% than I-100%, proving the toxin removal efficacy in the León WWTP. Liu et al. [42] evaluated several wastewater treatments in 96 hpf zebrafish larvae, showing a final 30% mortality reduction after each wastewater treatment stage, as expected. Interestingly, even the chemical compounds proven to be harder to remove in the effluent of León WWTP (carbamazepine and diclofenac) [44] showed less than 10% mortality at 24, 48, and 72 hpf in embryos exposed to 10^5^ times higher concentrations [45].

Beyond survival, hatching is considered a critical developmental endpoint, as it represents a sensitive indicator of sublethal stress during early embryogenesis. Although our experiments only revealed significant differences at 48 hpf between the control and E-75% condition and at 72 hpf between CTRL and E-100%, there was an overall trend in which both 100% influent and effluent reduced hatching rates, an effect that diminishes with dilution. This pattern may suggest a dose-dependent influence of compounds not fully removed during treatment. Previous works have only shown a decrease in hatching under 100% conditions compared to the control, and the higher the dilution tested for both I and E samples, the higher the hatching rate registered [13,41]. This could be explained by the role of the chorion, which influences the extent of chemical contact with embryos acting as a protective barrier [46,47], blocking the movement of molecules > 4 kDa [48]. However, the ability of toxins to cross the chorion is also affected by physicochemical properties, ionic charge, etc. [49], which may allow the uptake of these compounds, since the structure and permeability of the chorion change during development [50].

In line with these observations, morphological assessments revealed that the number of developmental malformations increased at 72 hpf in the influent group. At 120 hpf, the malformation rate increased in all experimental groups compared to the control (Figure 2E), being higher in the I group than in the E condition. Similarly, previous studies also reported an increase in the malformations rate at 24 and 48 hpf in similar samples to the ones we evaluated here [40,51] and at 96 hpf [42] and 144 hpf [41]. Since we observed a sharp increase in the incidence of malformations, we attempted to identify some of the possible causes of this increase, linking their appearance to the presence of nitrates [52], sulfates [53], metals like aluminum [54,55,56], or those pharmaceutical compounds that had lower removal efficiency in the Leon WWTP like the anti-inflammatory drug diclofenac [57] or the antiepileptic drug carbamazepine [45,58]. However, we failed to find a robust potential link since the previously reported values in the León WWTP [59] were much lower than those evaluated in the above-mentioned studies. Other studies evaluating the toxic effects of pesticides in 96 hpf larvae also reported an increase in malformations [60,61]; however, it is important to note that these specific pesticides, terbutryn and ethalfluralin, are no longer in use in Spain due to regulatory restrictions and environmental concerns. The type of malformations found in those studies and in others coincides with ours, including mainly pericardial edema, cardiac malformations, yolk sac edema, curvature of the spine, and absence of the swim bladder [13,41,42,51], as these are usually found in zebrafish exposed to compromising conditions. Therefore, many of these studies also performed heartbeat and biometric analyses [40,41,42,43,45].

#### 4.1.2. Heartbeat Analysis

Our heartbeat analysis used the *kop* transgenic line, which enabled direct visualization of the heart thanks to the *kop:mScarlet-I-nos 3′UTR*; *cmlc:GFP* construct. In this system, GFP expression is driven by the cardiac myosin light chain (*cmlc*) promoter specifically in cardiac tissue, causing the heart to appear green under fluorescence microscopy, which allowed us to accurately assess cardiac function and detect subtle alterations. We found a statistically significant decrease in the heartbeat in I-100% compared to CTRL. Similar heartbeat alterations have been previously reported in larvae exposed to wastewater [40,43,62]. Babic et al. [40] found an increased heartbeat in 48 hpf embryos in effluent water and no differences after the biological treatment [40], while Li et al. [43] reported a heartbeat reduction of 5% in effluent water. On the other hand, the experiments of Rothe et al. [62] showed an increase in heartbeat in 72 hpf larvae in the effluent, and Ribeiro et al. [41] reported similar results to ours, with a decreased heart rate in embryos exposed to I waters and no differences between E waters and control groups, proving the treatment’s efficacy in reducing cardiotoxic effects. Again, this group registered a concentration-dependent relation. It has also been shown that diclofenac at low concentrations can reduce the heart rate [57]; although in previous reports of the León WWTP the value was smaller and probably had no effect [44], it is possible that in the influent it had a higher concentration, and this is responsible for the decrease in the heart rate. It should also be noted that at 72 hpf we observed cardiac malformations in the influent, which could explain the reduction in heart rate detected in these larvae.

#### 4.1.3. PGC Number and Migration

In addition to these cardiovascular effects, we evaluated the migration of PGCs to the genital ridge, as proper PGC localization is crucial for reproductive development and is sensitive to environmental stressors. Previous studies have determined that the gonads are one of the primary targets of pollutants released in aquatic ecosystems, for example, 17alpha-ethinylestradiol. It was found that high doses of this compound resulted in altered migration and distribution of PGCs and the presence of ectopic PGCs in 20% of the embryos [63]. In our study, we found the highest percentage of aberrant embryos and delocalized PGCs in I-100% larvae as expected. This is in line with the quantified number of delocalized PGCs, although cluster length remained unchanged, suggesting that there could be certain compounds present in the influent water interfering with proper PGC migration. Several studies demonstrate that there are multiple endocrine disruptors released into water that can affect PGC number and distribution [64], triggering sterility [65] or affecting sexual differentiation [66,67]. Thus, these studies are of interest to determine whether the effluent water continues to have concentrations that interfere with the distribution or number of PGCs.

#### 4.1.4. Biometry Analysis

These developmental alterations were further reflected in biometric measurements. We performed these morphometric measurements to help eliminate subjective observations such as larvae length or pericardial edema as they make it possible to quantify changes that were not clear before. We reported a statistically significant decrease in the LL, MAL, and SL, as well as an increase in YSA and CA between all WWTP conditions compared to CTRL. Previous works also reported higher CA and YSA in 144 hpf larvae exposed to diluted influent and effluent waters [41]. Although biometry parameters like body length have been shown to be affected by wastewater even in young adults [68], other works reported no differences compared to the control in effluent-exposed 120 hpf larvae [62,69]. Thus, contradictory conclusions have been published in this regard, probably depending on the final chemical profile of each effluent. In addition, another study showed a dose-dependent axon length reduction in larvae exposed to neurotoxic compounds [70], showing a 20% decrease in the larval length [43]. In addition, the increase in cardiac area observed in I and E larvae supports the presence of these malformations and reinforces the link with the heartbeat alteration. As expected, diluting the water reduced the severity of biometric alterations, and effluent exposures generally caused fewer alterations than influent, suggesting a combined effect of water type and dose.

### 4.2. Behavioral Analysis

In addition to the phenotypic effects mentioned above, toxins can substantially modify the behavioral patterns of aquatic organisms due to the presence of neurotoxic compounds present in wastewater [71,72]. In the case of fish, behavior can be studied by analyzing the number of bursts or “tail coiling” within the chorion in embryos [73] and the swimming pattern in larvae [74]. In our experiment, the 24 hpf analysis revealed a decrease in the number of bursts/min in the influent probably due to some neurotoxic compound in the water capable of crossing the chorion [48,49]. This hypothesis is based on previous studies performed on 24 [62] and 48 hpf [40] larvae. Interestingly, our results showed higher differences at 120 hpf with lower motility values in all studied waters, with the exception of E75%. Again, these results provide evidence of a dose-dependent pattern. The sharp differences found at 120 hpf compared to 24 hpf embryos may be explained by the protective role of the chorion [46,47] and the accumulation of malformations affecting 120 hpf swimming ability.

### 4.3. Fin Regeneration Analysis

To further assess sublethal effects, we studied the tissue regeneration ability to better understand the impact of wastewater on zebrafish larvae physiology, as this species possesses the ability to regenerate many tissues and organs, including fins [75,76], the heart [77], and the spinal cord [78], among others. In particular, the fin is widely used to study regeneration due to its accessibility, simple structure, and quick regeneration [76,79]. In our study we found a decrease in regeneration ability in I- 100%, showing us that the chemical profile of the influent waters alters regeneration. Previous studies have proven that exposure to silver nanoparticles decreases regeneration and increases the abundance of neutrophils in the wound area [80], an observation that was also reported in 5 dpf larvae exposed to effluent waters [43]. On the other hand, other compounds proven to be found in wastewater influents like estrogen receptor antagonists, estrogenic endocrine-disrupting chemicals, and pesticides can also impair regenerative ability [81,82]. Therefore, studying this provides insight into the individual’s overall health and reflects the integration of a range of complex cellular processes, making it a highly informative endpoint in ecotoxicology.

### 4.4. Molecular Studies

Complementing these functional, behavioral, and morphological assessments, we also explored gene expression, as changes in the selected genes of our study could help elucidate the mechanisms underlying the observed developmental, behavioral, and regenerative alterations. We studied *hox* and *fox* genes, which are key regulators of body patterning during embryonic development and regulate key cellular and developmental processes, respectively [83,84,85]. Our gene expression results revealed a statistically significant downregulation of *hoxc6a* in both experimental groups, as well as a downregulation in *foxm1l* in I-100%. *hox* genes are among the earliest regulators of embryonic development, guiding axial mesoderm cell ingression during gastrulation [86]. Beyond this early role, Hox proteins are well established as transcriptional regulators critical for vertebrate hindbrain development by helping assign specific identities to emerging rhombomere segments [83]. In particular, *hoxc6a* contributes to both axial patterning and organ morphogenesis. Initially, it is expressed in the notochord with an anterior boundary around somite 2 and is later detected in the swim bladder primordium by 36 hpf [87]. Functionally, *hoxc6a* is essential for specifying anterior vertebral identity [84], and it was found that *hoxc6a* mutants displayed an abnormal anterior projection of the swim bladder beyond the fourth vertebra [88]. These findings indicate that *hoxc6a* not only patterns the vertebral column but also ensures correct positioning and structural development of the swim bladder. Interestingly, given the phenotypes found in our experiment, the bladder malformations could be related to the *hoxc6a* downregulation in the influent and effluent. Fox proteins are important regulatory transcription factors during development, especially for neuronal development, and are involved in multiple cellular processes, such as cell cycle progression, proliferation, differentiation, apoptosis, DNA damage response, and drug resistance [89,90,91]. Previous studies [92] reported the role of *foxm1*, revealing that its expression is increased in border zone cardiomyocytes, while *foxm1* mutants show decreased cardiomyocyte proliferation and expression of cell cycle genes, highlighting its role in regulating cell cycle checkpoints.

We also studied genes associated with proper development and canonical phenotype: *sox2*, whose knockout exhibits an uninflated swim bladder phenotype [93]; *ddx4*, which is specifically expressed in PGCs and plays a crucial role in their development, migration to the gonads, and maturation into functional gametes [94,95]; *hand2*, which regulates myocardial cells differentiation and is key in cardiac morphogenesis [13,96]; and *cenpf3b*, which is also involved in heart development [92]. However, we only found statistically significant differences in *cenpf3b*, showing a downregulation in the influent (Figure 8). Subsequent analysis of the centromere protein F, *cenpf*, a canonical target of *foxm1*, revealed that this microtubule and kinetochore binding protein is also required for heart regeneration. Moreover, *cenpf* mutants displayed elevated cardiomyocyte binucleation, indicating impaired mitosis. These findings demonstrate that both *foxm1* [97,98] and *cenpf* [99,100] are necessary for cardiomyocytes to complete cell division during zebrafish heart regeneration. Therefore, the downregulation we observed of these two genes in the influent may be one of the causes of the cardiac malformations found in the experiment and consequently of the reduced heart rate.

Between the ER-stress- and apoptosis-related genes (*ddit3* [101], *hspa5* [102], and *casp3* [103]), we only reported an overexpression of *ddit3* in I-100%. *ddit3* (DNA damage-inducible transcript factor 3) is a gene related to ER-stress-induced apoptosis [101]. Its overexpression together with the activating transcription factor 4 (*ATF4*) has been reported to lead to cell cycle arrest and/or apoptosis; as a transcriptional factor, it also has been shown to regulate numerous pro- and anti-apoptotic genes [101]. In relation to WWTPs, a previous study reported an overexpression of *ddit4* in the effluent [43], which is also induced by ER stress as a member of the same family as *ddit3*. This is in line with our findings, as we observed an overexpression of *ddit3* probably due to the presence of pollutants in the influent, generating ER stress and increasing mortality and malformations. Even though we did not find significant differences in *hspa5* (*p* = 0.0506), it is also interesting since it follows a similar trend and is also related to ER stress [104].

Genes related to dopamine and serotonin pathways were also analyzed. We did not find differences in the dopamine receptors, *drd1b* and *drd2a* [35,105], nor in the serotonin- and dopamine-pathway-related gene, *mao* [16]. However, our findings suggest a trend toward downregulation of *drd1b*, which may reflect alterations in dopaminergic signaling pathways, potentially affecting neurodevelopment and behavior in exposed larvae. A study by Tang et al. [105] demonstrated that the exposure of larvae to the antidepressant venlafaxine for 20 days led to the downregulation of *drd1b* and *drd2b*, accompanied by a reduction in locomotor activity. This finding is consistent with our behavioral observations, where we also detected alterations in larval motility. However, in our case, no significant differences were observed in the expression of dopamine receptor genes.

### 4.5. Dose–Effect Analysis

In addition to the different endpoints analyzed in our study—including survival, hatching, malformations, PGC migration, biometry, behavior, regeneration, and gene expression—the use of transgenic reporter lines such as *hsp70* represents an innovative tool to assess embryo responses to environmental stressors. To better reflect natural conditions in which effluent mixes with river water, we incorporated multiple dilutions in our dose–response analysis. Previous studies also used an *hsp70-EGFP* reporter gene to assess embryo stressors such as cadmium, proving EGFP translation responded in a dose-dependent manner, correlating with concentrations similar to those observed for morphologic indicators of early-life-stage toxicity [24]. This strongly supports our results since we observed fluorescence at those concentrations that are toxic to the embryo and are also accompanied by underdeveloped or aberrant phenotypes. The dose–effect experiments revealed significant differences between the CTRL group and the C^+^, I-100%, I-75%, and E-100% groups, showing an increase in malformations and correlating with a dose-dependent effect of chemicals in the water, as discussed above with the *kop* transgenic line and other studies [40,41,42]. We also registered statistically significant differences in the amount of 24 hpf embryo tail coiling between some of the groups (Figure 10B). In contrast, the number of bursts/min did not seem to follow this dose-dependent pattern but rather the type of analyzed water. Nevertheless, two different patterns can be distinguished: a lower activity in the influent-exposed larvae and a higher number of bursts in those exposed to the effluent compared to the control. Therefore, we hypothesize that the influent water has more neurotoxic compounds, while the effluent water has more neurostimulant compounds.

In summary, our study demonstrates that zebrafish embryos and larvae prove to be a sensitive and versatile model for assessing multiple endpoints using transgenic reporter lines. The dose- and water-type-dependent effects observed highlight the capacity of this model to detect subtle sublethal impacts of wastewater, which may not be apparent through conventional chemical analyses alone. Integrating this model as a complementary test in wastewater treatment plants can offer a more comprehensive evaluation of water quality, supporting better environmental monitoring and protection of aquatic ecosystems.

## 5. Conclusions

Zebrafish embryos and larvae proved to be a sensitive and reliable model for evaluating water quality, revealing subtle developmental and behavioral effects from environmental exposures. The secondary treatment process of the León WWTP markedly reduced toxicity, as shown by the lower incidence and severity of effects in organisms exposed to treated versus untreated water. The use of the *hsp70* transgenic line confirmed that effects observed in undiluted effluent disappeared upon dilution, mimicking natural conditions after discharge into the river. These findings highlight the zebrafish model as a valuable tool for environmental monitoring and demonstrate the importance of current treatment protocols in protecting aquatic life.

### Specific Conclusions

1. Zebrafish embryos and larvae exposed to both influent and effluent waters exhibited high overall survival rates (>90%). However, statistically significant differences were detected, suggesting subtle effects on viability. Delays in hatching were observed at 48 hpf in embryos exposed to 75% effluent and at 72 hpf in those exposed to 100% effluent.
2. Exposure to both influent and effluent waters resulted in significant morphological alterations, particularly affecting biometry as well as malformed swim bladder, poor yolk sac reabsorption, yolk sac and pericardial edema, and heart malformations. Notably, the heart rate was significantly altered only in embryos exposed to 100% influent. Behavioral analysis revealed a significant reduction in the number of bursts in embryos exposed to 100% influent and in larvae motility in all wastewater-exposed groups, except in the 75% effluent condition, which was comparable to the control.
3. Exposure to 100% influent (I-100%) significantly increased the number of delocalized PGCs, while no differences were observed in cluster length. In contrast, effluent exposure did not produce significant alterations in PGC migration or localization.
4. The regenerative capacity of the caudal fin was only affected in larvae exposed to 100% influent water.
5. Exposure to influent water caused a significant downregulation of *foxm1l* and *cenpf3b*, while *hoxc6a* was downregulated in both influent and effluent conditions. In contrast, *ddit3* was overexpressed. This suggests that influent water exerts a negative impact on key genes involved in axial and cardiac development, potentially explaining the observed malformations and reduced heart rate, and in genes related to ER stress and apoptosis.
6. The use of the *hsp70* transgenic reporter line indicated that malformations observed at 24 hpf were no longer present when embryos were exposed to diluted effluent. Embryos exposed to effluent water showed increased spontaneous movement within the chorion compared to controls.

## Figures and Tables

**Table 1:** Primers used for the mRNA analysis. Gene name, accession number, primer sequences, product size (bp), melting temperature (Tm; °C), and efficiency (%) are represented.

Gene Name	Accession Number	Oligo	Sequence (5′ to 3′)	Product Size (bp)	Melting Temperature (°C)	Efficiency (%)	Ref.
*actb2*	NM_181601.5	F	CGTGCTGTCTTCCCATCCA	86	60	108.62	[32]
		R	TCACCAACGTAGCTGTCTTTCTG				
*rps18*	NM_173234.1	F	AACACGAACATTGATGGAAGACG	255	60	96.16	[33]
		R	ATTAGCAAGGACCTGGCTGTATTT				
*mao*	NM_212827.3	F	ACCAACTCAAAACCGCATTC	151	60	97.21	[34]
		R	GTAGGCAAAAGGGTTCCACA				
*hand2*	BC083365.1	F	ATGAGTTTAGTTGGAGGGTTTC	324	62	108.56	[13]
		R	GCTGTTGATGCTCTGGGT				
*drd2a*	NM_183068.1	F	TGTGATTGCGAATCCTGCCT	194	60	104.83	[35]
		R	CGGGATGGGTGCATTTCTTT				
*cenpf3b*	XM_002665215.7	F	AAACGGCACTGACAAGTTGG	380	60	103.29	[36]
		R	GCCCACCTTCTGCCATAGTT				
*casp3*	NM_131877.3	F	GGCAGATTTCCTCTATGCATACTC	72	60	98.30	[37]
		R	CATGAGCCGGTCATTGTG				
*hspa5*	NM_213058.1	F	AAGAGGCCGAAGAGAAGGAC	133	60	104.63	
		R	AGCAGCAGAAGCCTCGAAATA				
*ddit3*	NM_001082825.1	F	AAGGAAAGTGTAGGAGCTGA	197	60	94.39	
		R	TCACGCTCTCCACAAGAAGA				
*sox2*	NM_213118.1	F	ACTCCATGACCAACTCGCAG	159	60	97.86	[38]
		R	AATGAGACGACGACGTGACC				
*ddx4*	NM_131057.1	F	ATGGCATTCCCATCATTTCAG	74	60	107.50	
		R	GGCCGCCGTTTTTCCT				
*drd1b*	NM_001135976.2	F	ACGCTGTCCATCCTTATCTC	135	60	105.50	This work
		R	TGTCCGATTAAGGCTGGAG				
*foxf1*	NM_001080186.1	F	TGCACGGGATCATCAGGGAC	113	60	90.47	
		R	GCCGAGGCCGTGCTAGAATA				
*foxk1*	NM_199902.1	F	TGAACCAGGAAGCCAGCGAA	173	60	90.39	
		R	ACATTCGATCAGGTGCCCGT				
*foxl1*	NM_200984.1	F	GTCTCCCTCCCGAGATGCAC	115	60	90.64	
		R	CACTCTTTACGGGCACACGC				
*foxm1l*	NM_201097.1	F	CGACCAGAAGCAAACCGCTG	84	60	99.71	
		R	GATCTGAGGGCAAGTGGGGG				
*foxq1a*	NM_001243344.1	F	GATCCTTCGAGACCGTGGGG	187	60	106.65	
		R	TCGAAGGAGGCGTAGCGATG				
*hoxa3a*	NM_131534.2	F	GGCCAGCTCTTGGTTTACCC	169	60	101.84	
		R	TGTAAATTGCCGAGCCGTCG				
*hoxc4a*	NM_131122.2	F	AGCTCAGCCTCTGCCAAACA	97	60	92.87	
		R	GCTTGGGTTCCGCTCCATTG				
*hoxc6a*	NM_131123.1	F	CCACGTTGCCCAGGAGTACA	114	60	90.60	
		R	ACTCCGCTGTGCGAGTTCAT				
*hoxc8a*	NM_001005771.1	F	GGCGGCGAAACATTAGAGCC	197	60	107.82	
		R	GCCAATGCACAGGGGTTCTG				

## Data Availability

All data supporting the findings of this study are available within the article.

## Abbreviations

The following abbreviations are used in this manuscript:

At	Atrium
AVC	Atrioventricular canal
BOD_5_	Biochemical Oxygen Demand over five days
CA	Cardiac area
*cenpf*	Centromere protein F
Cmlc	Cardiac myosin light chain
COD	Chemical Oxygen Demand
C^+^	Positive control
CTRL	Control
*ddit3*	DNA Damage Inducible Transcript 3
Dpa	Days post-amputation
Dpf	Days post-fertilization
DSS	Decision Support Systems
E	Effluent water from the secondary treatment in the León WWTP (different concentrations of effluent water are named E-100% and E-75%)
EM	Embryo medium
Endoc	Endocardium
ER	Endoplasmic reticulum
*foxm1*	*forkhead box M1*
*hoxc6*	*homeobox C6*
Hpf	Hours post-fertilization
*Hsp70*	Tg(hsp70l:dn-fgfr1a-EGFP)
I	Influent water to the secondary treatment in the León WWTP (different concentrations of influent water are named I-100% and I-75%)
*Kop*	Tg(kop:mScarlet-I-nos 3′UTR-cmlc:GFP)
LL	Larvae length
MAL	Mouth-to-anus length
Myoc	Myocardium
PBS	Phosphate-buffered saline
PGCs	Primordial germ cells
ROI	Region of interest
SEM	Standard error of the mean
SL	Spine length
V	Ventricle
WWTP	Wastewater treatment plant
YSA	Yolk sac area
